# Serum untargeted metabolomic changes in response to diet intervention in dogs with preclinical myxomatous mitral valve disease

**DOI:** 10.1371/journal.pone.0234404

**Published:** 2020-06-18

**Authors:** Qinghong Li, Dorothy P. Laflamme, John E. Bauer

**Affiliations:** 1 Nestlé Purina Research, St. Louis, MO, United States of America; 2 Scientific Communications Consultant, Floyd, VA, United States of America; 3 Professor Emeritus, Texas A&M University, Longmont, CO, United States of America; Universite de Liege (B34), BELGIUM

## Abstract

Myocardial energy deprivation plays a causal role in the development of heart failure. A cardiac protection blend (CPB) of nutrients including medium chain triglycerides, fish oil and other key nutrients was developed to slow the progression of canine myxomatous mitral valve disease (MMVD). A six-month dietary intervention demonstrated efficacy of CPB in slowing MMVD progression. Untargeted metabolomic analysis of serum from these dogs identified 102 differential metabolites (adjusted P < 0.05). The ratios of omega-6 to omega-3 fatty acid (FA) changed from 2.41 and 1.46 in control and CPB groups at baseline to 4.30 and 0.46 at 6 months respectively. A 2.7-fold increase of α-aminobutyrate, a myocardial modulator of glutathione homeostasis, was found in CPB dogs compared to 1.3-fold increase in control dogs. Arginine and citrulline, precursors of nitric oxide biosynthesis, were both increased 2-fold; caprate, a medium chain FA, was increased 3-fold; and deoxycarnitine, precursor of carnitine biosynthesis, was increased 2.5-fold in CPB dogs. Margarate and methylpalmitate decreased in response to CPB, a potential benefit in MMVD dogs as positive correlations were found between changes in both these FAs and left atrial diameter (r = 0.69, r = 0.87 respectively, adjusted P < 0.05). Sphingomyelins with very long chain saturated FAs associated with decreased risk of heart failure in humans were increased in MMVD dogs fed the CPB diet. Our data supports the hypothesis that CPB improves FA utilization and energetics, reduces oxidative stress and inflammation in MMVD dogs. More studies are needed to understand the roles of specific metabolites in MMVD.

## Introduction

The adult mammalian heart requires a large quantity of ATP produced through mitochondrial fatty acid (FA) oxidation in order to support its normal contractile work [1]. Perturbations in myocardial energy metabolism play a key role in the development of heart failure [2–4]. A shift from long chain fatty acids (LCFAs) as the main energy source to other energy substrates has been documented in the failing heart in both humans and animals [5–7]. Chronic myxomatous mitral valve disease (MMVD) is the most common naturally-occurring heart disease in dogs affecting approximately 10–15% of the canine population, with greater frequency in geriatric, small, or medium breed dogs [8]. It is characterized by a slowly progressive valvular degeneration that can cause mitral regurgitation and ultimately lead to congestive heart failure.

Gene expression and metabolomic profiling studies comparing healthy dogs and dogs with MMVD revealed alterations in energy metabolism, oxidative stress, inflammation and extracellular matrix homeostasis [9–11]. Similar findings were documented in human MMVD, which afflicts 2–3% of the general population [12–16]. Canine MMVD is considered a model for MMVD in man [13, 16]. To address those metabolic changes in dogs, a cardiac protection blend (CPB) of nutrients was formulated to include medium chain triglycerides (MCT) as an alternative energy source, carnitine precursors to enhance FA oxidation, fish oil to reduce inflammatory mediators, antioxidants to reduce oxidative stress, and magnesium to support myocardial function [17].

Medium chain fatty acids (MCFAs) from MCTs do not require transporters or carnitine-mediated transport pathway to reach mitochondria and thus can be rapidly oxidized for energy [18, 19]. A MCT-supplemented diet has been shown to prevent progressive cardiac remodeling in spontaneously hypertensive rats, possibly by maintenance of myocardial energy and reduction in oxidative stress [20]. MCTs have been proposed for potential clinical application in the management of cardiovascular diseases in humans [21, 22]. In dogs, a 6-month blinded, randomized placebo-controlled dietary intervention study with this CPB demonstrated beneficial effects in reducing left atrial enlargement and in slowing or preventing the progression of early stage MMVD [17]. The objective of the study presented here was to evaluate the serum metabolomic changes associated with this dietary supplement and with the clinical improvement observed in these dogs.

The metabolome encompasses a large variety of metabolites, the small intermediary molecules in metabolism. Metabolomics has been increasingly used to investigate metabolic pathways in cardiovascular diseases [23–27], including mitral valve disease in both dogs [9] and humans [15]. Emerging evidence points to derangements in cardiac metabolism including perturbations in mitochondrial bioenergetics and reactive oxygen species (ROS) homeostasis in failing hearts [1]. The hypothesis of this study was that serum metabolomic changes in response to the CPB diet would provide markers reflecting improved metabolic states in mitochondrial bioenergetics, oxidative stress and inflammation in dogs with early stage MMVD. These findings would advance understanding in MMVD pathogenesis and modulation of cardiac metabolism could provide potential alternative therapies for MMVD in both humans and dogs.

## Materials and methods

### Diets, animals, and study design

The study design of this blinded, randomized, placebo-controlled study, including detail on the diets, animals, husbandry and sampling protocols, was previously published [17]. The two study diets (control (CON) and CPB) were similar in protein, total fat, and carbohydrate contents: the MCTs and fish oil in the CPB diet were balanced with animal fats in the CON diet [17]. Additional analysis of dietary amino acids and fatty acids is provided in S1 Table. Nineteen small to medium-sized dogs (age range: 7.9–13.7 years) with naturally-occurring preclinical MMVD were enrolled from among the permanent canine population of a Nestlé Purina PetCare Center. All dogs were fed the same commercial diet, with a nutrient profile similar to the CON diet, during the baseline period; then received either the CON (n = 9) or CPB (n = 10) diet for the duration of the 6-month study. One dog in the CON group developed an illness unrelated to the diet after 3 months and was excluded from this study. After completion of this study, dogs were returned to the general canine population in the Nestlé Purina PetCare Center. The study protocol was approved by the Institutional Animal Care and Use Committee of the Nestlé Purina PetCare Company.

### Serum samples and metabolomics assay

Venous blood samples were collected from each dog after overnight fasting at baseline and 6 months. Six milliliters of blood were drawn by jugular venipuncture into serum separator tubes and allowed to separate for 30 minutes in room temperature. The tubes were then centrifuged at 3500 RPM for 9 minutes. Aliquots of 500 microliters serum from the clear top layer were transferred into small Cryovials and stored in -80°C until analyzed. Serum samples were shipped on dry ice to Metabolon, Inc. (Durham, NC) for sample processing and untargeted metabolomics assays. Sample preparation and extraction, liquid chromatography and mass spectrometry methods, and compound detection and identification were performed by Metabolon Inc. using Metabolon’s standard protocols and software [28] (S1 File). A total of 759 metabolites were identified, including 619 known and 140 unknown.

### Pre-processing of metabolomics data

The raw data was based on area-under-the-curve generated using ion counts that provide relative quantification (S2 File). Metabolites with a constant value across all samples or with more than 80% of missing data were removed. Under the assumption that missing data were values below the detection limit, the remaining missing values were estimated and replaced by a value equal to half of the minimal positive value in the original data [29]. The data were further processed using generalized logarithm transformation [30],

$$g\log_{2}\left(x\right)=\log_{2}\frac{x+\sqrt{\left(a^{2}+x^{2}\right)}}{2}$$

where *a* is a constant with a default value of 1. Finally, transformed data was auto-scaled to achieve a zero mean and unit variance for any variable: $z_{ij}=\frac{x_{ij}-\bar{x_{i}}}{s_{i}}$ where *x_ij_* is the value of the *i*^*th*^ metabolite and *j*^*th*^ sample, and $\bar{x_{i}}$ *and s_i_* are the mean and standard deviation respectively. The advantage of autoscaling is that all metabolites become equally important [31].

### Statistical analysis

Principal component analysis (PCA) for high-dimensional multivariate data was performed on the baseline and 6-month data. The first two principal components (PCs) which capture more data variations than other PCs were examined for their ability to separate groups based on diet (CON vs CPB) or time (0 vs 6 months). A multiple linear regression using each PC as the dependent variable and diet or time as the independent variable, adjusted for sex, age, breed, and body condition score (BCS) was performed and the P-value for the model was obtained. The 6-month data were subjected to partial least-squares discriminant analysis (PLS-DA) to detect key metabolites with the highest Variable Importance in Projection (VIP) scores. A maximum of five components were searched for optimal performance and leave-one-out cross-validation was performed to assess model’s predictive accuracy.

For each metabolite, the effect of diet by time interaction was estimated using a linear mixed-effect model adjusted for age, breed, sex, and BCS. P-values for the interaction terms were obtained and adjusted for multiple testing to control false discovery rate (FDR) [32]. A metabolite was considered significant if FDR was less than 0.05. Fold change (FC) is defined as the ratio of 6 months (*t*6) over baseline (*t*0). Because the minimal value in the raw data is far greater than 1, *FC* ≈ 2^(*t*6−*t*0)^ *if t*6 ≥ *t*0 or $FC\approx \frac{-1}{2^{\left(t6-t0\right)}}$ if *t*6 < *t*0 *s*uch that a negative FC indicates a decrease in value from baseline to 6 months. Spearman’s correlation analysis was performed on the changes from baseline to 6 months between left atrial diameter (LAD) and significant metabolites. A subsequent analysis was performed to assess the correlations between methylpalmitate, margarate, and other significant metabolites. P-values were adjusted for multiple testing. The ratio of omega-6 to omega-3 FAs was determined as the sum of six omega-6 FAs (linoleate (18:2n6), arachidonate (20:4n6), dihomolinoleate (20:2n6), docosadienoate (22:2n6), adrenate (22:4n6), and n6-DPA (22:5n6)) divided by the sum of four omega-3 FAs (linolenate (18:3n3), EPA (20:5n3), DPA (22:5n3), and DHA (22:6n3)). Heatmaps of phospholipids and sphingomyelins were generated using the changes from baseline to 6 months from each dog. Metabolomics data processing and statistical analysis were performed using MetaboAnalyst 3.0, a comprehensive tool suite for metabolomics data analysis [29] and the statistical computing software R (version 3.5.0) [33].

## Results

### Dietary effects on metabolome

**Fig 1:**
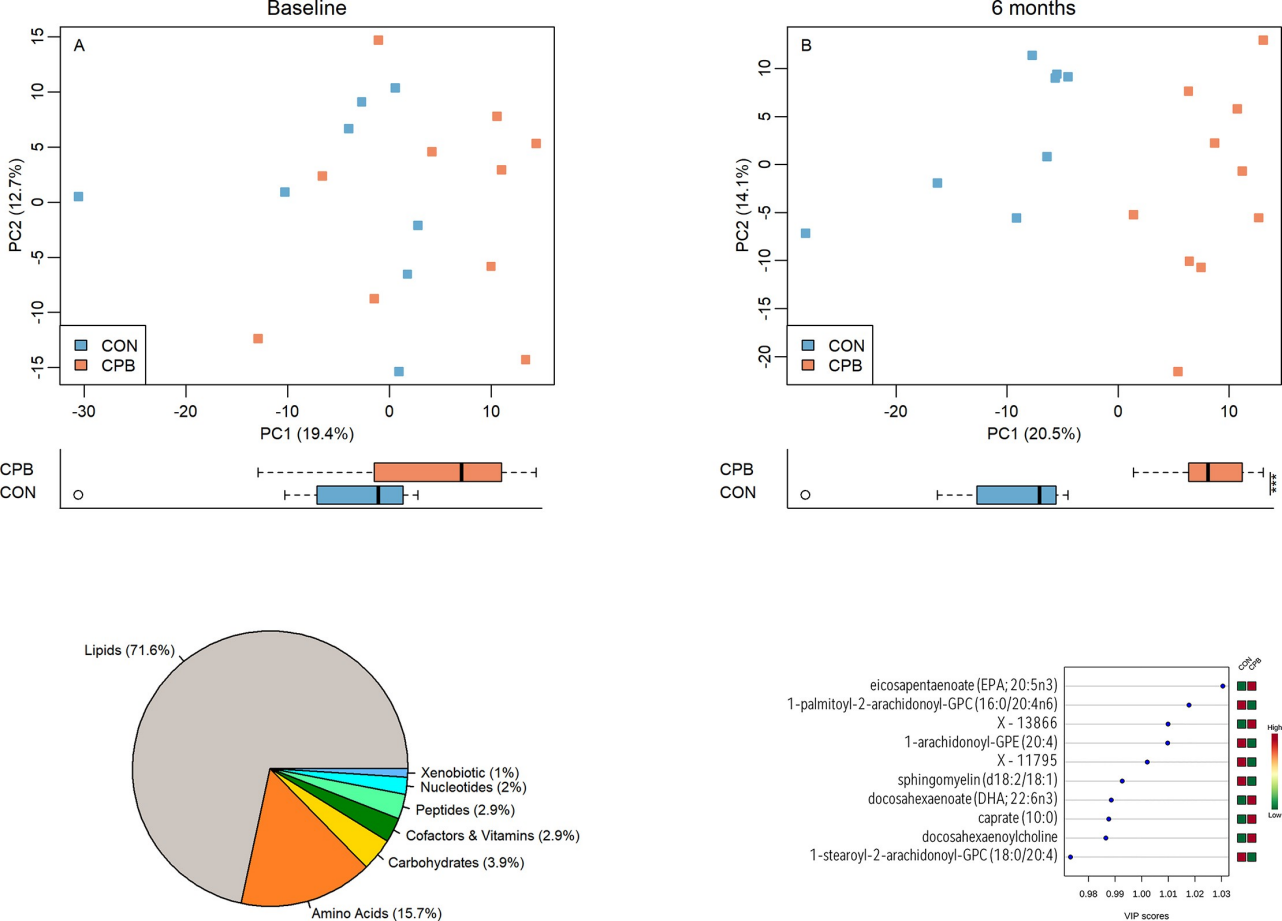
(A, B) Principal component analysis (PCA) based on diet effects at baseline (A) and 6 months (B). The percentages of data variation explained by the first two principal components, PC1 and PC2, are indicated on the x and y axes respectively. Distributions of samples along PC1 by diets were plotted below each PCA plot. Blue squares represent CON diet while orange ones represent CPB diet. *P < 0.05; **P < 0.01; ***P < 0.001. (C) Pie chart shows percentage of each metabolite class. (D) Partial least squares discriminant analysis (PLS-DA) identified ten metabolites with the highest Variable Importance in Projection (VIP) scores, which indicates discriminant power between groups.

Principal component analysis (PCA) showed no significant clustering at baseline, but clustering by diet along PC1 became evidenced at 6 months (P = 0.19, P = 1.54e-04 respectively, Fig 1A and 1B). The p-values and effects of the adjustment for sex, age, breed, and BCS from the multiple regression models were reported in S3 File. The first two principal components, PC1 and PC2, account for 19.4% and 12.7% of the total data variation at baseline and 20.5% and 14.1% at 6 months respectively. PCA performed to evaluate changes over time found no change in CON dogs but significant time effect along PC1 was observed in CPB dogs ((P = 0.74, P = 3.4e-04 respectively, S1 Fig). No separation was found along PC2 (all P > 0.05).

Linear mixed model analysis identified 112 metabolites that were significant in diet by time interaction (all FDR < 0.05, Table 1). Among 102 known metabolites, 73 were lipids (71.6%) and 16 were amino acids (15.7%) (Fig 1C).

**Table 1:** Metabolites with significant diet by time interaction.

Metabolites	Class	Pathway	P	FDR	FC_CON	FC_CPB	KEGG	PUBCHEM	HMDB
carboxyethyl-GABA	AA	Glu Metabolism	0.0036	0.0284	-1.22	-1.8		2572	02201
Lysine	AA	Lys Metabolism	0.0023	0.0211	1.14	2.37	C00047	5962	00182
2-aminoadipate	AA	Lys Metabolism	0.0056	0.0378	-1.46	2.32	C00956	469	00510
Phenylalanine	AA	Phe and Tyr Metabolism	0.0015	0.0147	-1.53	2.8	C00079	6140	00159
N-acetylphenylalanine	AA	Phe and Tyr Metabolism	0.0009	0.0105	1.02	2.16	C03519	74839	00512
5-bromotryptophan	AA	Try Metabolism	0.0048	0.034	-1.02	-1.64		96735	
N-acetylleucine	AA	Leu, Ile and Val Metabolism	0.007	0.0456	-1.06	1.5	C02710	70912	11756
S-methylmethionine	AA	Met, Cys, SAM and Tau Metab	0.0006	0.0082	-1.12	2.29	C05319	458	38670
methionine sulfone	AA	Met, Cys, SAM and Tau Metab	0.0074	0.0471	-1.36	-3.41		69961	
Cystathionine	AA	Met, Cys, SAM and Tau Metab	0.0002	0.0037	-1.51	3.13	C02291	439258	00099
S-methylcysteine	AA	Met, Cys, SAM and Tau Metab	0.0008	0.0101	1.52	2.92		24417	02108
Hypotaurine	AA	Met, Cys, SAM and Tau Metab	0.0022	0.0199	-1.27	1.86	C00519	107812	00965
Taurine	AA	Met, Cys, SAM and Tau Metab	0.0048	0.034	1.29	3.89	C00245	1123	00251
Arginine	AA	Urea cycle; Arg and Pro Metab	0.0042	0.0314	-1.49	2.09	C00062	232	00517
Citrulline	AA	Urea cycle; Arg and Pro Metab	0.0028	0.0239	-1.01	2.09	C00327	9750	00904
2-aminobutyrate	AA	Glutathione Metabolism	0.0031	0.0255	1.29	2.69	C02261	439691	00650
gamma-glutamyl-alpha-lysine	Peptide	Gamma-glutamyl amino acid	0.0017	0.0163	1	2.57		65254	
gamma-glutamylphenylalanine	Peptide	Gamma-glutamyl amino acid	0.0011	0.0127	-1.37	2.37		111299	00594
Anserine	Peptide	Dipeptide Derivative	0.0013	0.0133	-2.12	-1.08	C01262	112072	00194
Glycerate	CHO	Glycolysis, Gluconeogenesis & Pyruvate Metabolism	0.003	0.026	-1.59	1.33	C00258	752	00139
Ribitol	CHO	Pentose Metabolism	0.0065	0.0436	-1.51	1.62	C00474	6912	00508
2-ketogulonate	CHO	Fructose, Mannose & Galactose Metabolism	0.004	0.031	-1.09	2.26	C15673	50	11732
N-acetylneuraminate	CHO	Aminosugar Metabolism	0.0051	0.0352	1.6	3.44	C00270	439197	00230
caprate (10:0)+A25:J39	Lipid	Medium Chain Fatty Acid	0	0.0002	1.03	3.01	C01571	2969	00511
margarate (17:0)	Lipid	Long Chain Fatty Acid	0.004	0.0304	1.17	-2.31		10465	02259
10-heptadecenoate (17:1n7)	Lipid	Long Chain Fatty Acid	0.0015	0.0147	1.3	-2.49		5312435	60038
oleate/vaccenate (18:1)	Lipid	Long Chain Fatty Acid	0.0043	0.0315	1.28	-2.19			
eicosapentaenoate (EPA; 20:5n3)	Lipid	Polyunsaturated FA (n3 & n6)	0	0	-1.49	3.07	C06428	446284	01999
docosapentaenoate (DPA; 22:5n3)	Lipid	Polyunsaturated FA (n3 & n6)	0.0003	0.0046	-1.34	2.71	C16513	6441454	06528
docosahexaenoate (DHA; 22:6n3)	Lipid	Polyunsaturated FA (n3 & n6)	0.0002	0.0033	-1.72	2.32	C06429	445580	02183
arachidonate (20:4n6)	Lipid	Polyunsaturated FA (n3 & n6)	0.0021	0.0198	1.36	-2.25	C00219	444899	01043
adrenate (22:4n6)	Lipid	Polyunsaturated FA (n3 & n6)	0.0002	0.0037	1.62	-2.12	C16527	5497181	02226
docosapentaenoate (n6 DPA; 22:5n6)	Lipid	Polyunsaturated FA (n3 & n6)	0.0004	0.006	1.97	-1.89	C16513	6441454	01976
mead acid (20:3n9)	Lipid	Polyunsaturated FA (n3 & n6)	0.0012	0.0127	1.29	-2.88		5312531	10378
methylpalmitate (15 or 2)	Lipid	FA, Branched	0.0007	0.0082	1.1	-2.76		17903417	
sebacate (decanedioate)	Lipid	FA, Dicarboxylate	0.0013	0.0133	1.49	2.75	C08277	5192	00792
octadecanedioate (C18)	Lipid	FA, Dicarboxylate	0.0067	0.0446	1.31	-1.8		70095	00782
Eicosanodioate	Lipid	FA, Dicarboxylate	0.0022	0.0199	1	2.48		75502	
Docosadioate	Lipid	FA, Dicarboxylate	0.0002	0.0036	-1.11	2.87	C19625	244872	
oleoylcarnitine (C18)	Lipid	FA Metab(Acyl Carnitine)	0.0038	0.0295	1.03	-2.41		6441392	05065
adipoylcarnitine (C6-DC)	Lipid	FA Metab(Acyl Carnitine)	0.0027	0.0236	1.13	-1.74		71296139	61677
margaroylcarnitine	Lipid	FA Metab(Acyl Carnitine)	0.0012	0.0128	-1.04	-2.62			06210
Deoxycarnitine	Lipid	Carnitine Metab	0.0002	0.0037	-1.11	2.51	C01181	134	01161
glycerophosphoethanolamine	Lipid	Phospholipid Metabolism	0.008	0.0486	2.15	-1.23	C01233	123874	00114
1,2-dipalmitoyl-GPC (16:0/16:0)	Lipid	Phospholipid Metabolism	0	0.0006	-1.85	2.54	D03585	452110	00564
1-palmitoyl-2-oleoyl-GPC (16:0/18:1)	Lipid	Phospholipid Metabolism	0	0.0007	-1.3	-3.45		6436017	07972
1-palmitoyl-2-linoleoyl-GPC (16:0/18:2)	Lipid	Phospholipid Metabolism	0	0.0003	1.02	-3.16		5287971	07973
1-stearoyl-2-arachidonoyl-GPC (18:0/20:4)	Lipid	Phospholipid Metabolism	0.0004	0.0059	1.25	-2.16		16219824	08048
1-palmitoyl-2-arachidonoyl-GPC (16:0/20:4n6)	Lipid	Phospholipid Metabolism	0	0.0001	1.12	-3.35		10747814	07982
1-linoleoyl-2-linolenoyl-GPC (18:2/18:3)	Lipid	Phospholipid Metabolism	0	0	-1.34	3.3			08141
1-palmitoyl-2-palmitoleoyl-GPC (16:0/16:1)	Lipid	Phospholipid Metabolism	0	0.0009	-1.22	-3.32			07969
1-palmitoyl-2-arachidonoyl-GPE (16:0/20:4)	Lipid	Phospholipid Metabolism	0.0002	0.0035	1.01	-3.65		9546800	05323
1-palmitoyl-2-stearoyl-GPC (16:0/18:0)	Lipid	Phospholipid Metabolism	0.0078	0.0477	-1.61	1.97			07970
1-palmitoleoyl-2-linoleoyl-GPC (16:1/18:2)	Lipid	Phospholipid Metabolism	0.0007	0.0084	1.28	-1.94			08006
1-linoleoyl-2-arachidonoyl-GPC (18:2/20:4n6)	Lipid	Phospholipid Metabolism	0.0006	0.0082	-1.25	2.22			08147
docosahexaenoylcholine	Lipid	Phospholipid Metabolism	0.0027	0.0236	-1.02	2.95			
1-palmitoyl-2-gamma-linolenoyl-GPC (16:0/18:3n6)	Lipid	Phospholipid Metabolism	0	0.0009	1.08	-3.4			07974
1-palmitoyl-GPC (16:0)	Lipid	Lysolipid	0.0013	0.0133	1.65	-2.1		86554	10382
1-linoleoyl-GPC (18:2)	Lipid	Lysolipid	0.0053	0.0367	1.38	-2.34	C04100	11988421	10386
1-arachidonoyl-GPC (20:4)	Lipid	Lysolipid	0.0002	0.0036	1.83	-2.38	C05208		10395
1-lignoceroyl-GPC (24:0)	Lipid	Lysolipid	0.0002	0.0037	-1.13	3.17			10405
1-oleoyl-GPE (18:1)	Lipid	Lysolipid	0.0035	0.0278	1.27	-2.54		9547071	11506
1-linoleoyl-GPE (18:2)	Lipid	Lysolipid	0.0002	0.0033	1.24	-2.66		52925130	11507
1-arachidonoyl-GPE (20:4)	Lipid	Lysolipid	0	0	1.29	-3.54		42607465	11517
1-arachidonoyl-GPI (20:4)	Lipid	Lysolipid	0.0028	0.0242	1.6	-1.95			61690
1-(1-enyl-palmitoyl)-2-palmitoyl-GPC (P-16:0/16:0)	Lipid	Plasmalogen	0.0006	0.0077	-1.28	1.23		11146967	11206
1-(1-enyl-palmitoyl)-2-arachidonoyl-GPE (P-16:0/20:4)	Lipid	Plasmalogen	0	0.0003	1.25	-3.17			11352
1-(1-enyl-stearoyl)-2-oleoyl-GPE (P-18:0/18:1)	Lipid	Plasmalogen	0.0001	0.0026	1.03	-2.7			11375
1-(1-enyl-palmitoyl)-2-arachidonoyl-GPC (P-16:0/20:4)	Lipid	Plasmalogen	0	0	1.15	-3.43			11220
1-(1-enyl-palmitoyl)-2-linoleoyl-GPC (P-16:0/18:2)	Lipid	Plasmalogen	0	0	-1.02	-3.07			11211
1-(1-enyl-stearoyl)-2-arachidonoyl-GPE (P-18:0/20:4)	Lipid	Plasmalogen	0	0.0004	1.17	-2.59		9547058	05779
myristoyl dihydrosphingomyelin (d18:0/14:0)	Lipid	Sphingolipid Metabolism	0.0039	0.03	-1.53	1.62			12085
palmitoyl SM (d18:1/16:0)	Lipid	Sphingolipid Metabolism	0.0009	0.0111	-1.24	-3.14		9939941	
SM (d18:2/14:0, d18:1/14:1)	Lipid	Sphingolipid Metabolism	0.0001	0.0023	-1.47	2.36			
SM (d18:2/16:0, d18:1/16:1)	Lipid	Sphingolipid Metabolism	0	0.0004	-1.09	-3.27			
SM (d18:1/18:1, d18:2/18:0)	Lipid	Sphingolipid Metabolism	0	0.0001	-1.34	-4.1		6443882	12101
SM (d18:1/20:0, d16:1/22:0)	Lipid	Sphingolipid Metabolism	0	0.0007	-1.59	2.06			12102
SM (d18:1/20:1, d18:2/20:0)	Lipid	Sphingolipid Metabolism	0.0076	0.0473	-1.23	1.64			
SM (d18:1/21:0, d17:1/22:0, d16:1/23:0)	Lipid	Sphingolipid Metabolism	0.0011	0.0127	-1.57	1.97			
SM (d18:1/22:1, d18:2/22:0, d16:1/24:1)	Lipid	Sphingolipid Metabolism	0.0001	0.0025	-1.63	1.6			12104
SM (d18:2/23:0, d18:1/23:1, d17:1/24:1)	Lipid	Sphingolipid Metabolism	0.0029	0.0245	-1.54	1.79			
SM (d18:1/24:1, d18:2/24:0)	Lipid	Sphingolipid Metabolism	0.0005	0.0064	-1.54	1.93			12107
SM (d18:2/24:1, d18:1/24:2)	Lipid	Sphingolipid Metabolism	0.0002	0.0037	-1.44	1.82			
N-stearoyl-sphingosine (d18:1/18:0)	Lipid	Sphingolipid Metabolism	0.0054	0.0367	-1.2	-3.07		5283565	04950
N-stearoyl-sphingadienine (d18:2/18:0)	Lipid	Sphingolipid Metabolism	0.0069	0.045	-1.23	-3.21			
lactosyl-N-nervonoyl-sphingosine (d18:1/24:1)	Lipid	Sphingolipid Metabolism	0.0013	0.0133	-1.75	1.76			
SM (d18:2/23:1)	Lipid	Sphingolipid Metabolism	0	0	-1.96	2.14			
SM (d18:2/21:0, d16:2/23:0)	Lipid	Sphingolipid Metabolism	0	0.0001	-1.63	2.27			
SM (d18:2/24:2)	Lipid	Sphingolipid Metabolism	0	0.0003	-1.29	1.86			
SM (d18:1/22:2, d18:2/22:1, d16:1/24:2)	Lipid	Sphingolipid Metabolism	0	0.0002	-1.42	1.93			
SM (d18:0/18:0, d19:0/17:0)	Lipid	Sphingolipid Metabolism	0.0002	0.0033	-1.32	-3.54			12087
SM (d18:2/18:1)	Lipid	Sphingolipid Metabolism	0	0	-1.11	-4.62			
SM (d18:1/19:0, d19:1/18:0)	Lipid	Sphingolipid Metabolism	0	0.0001	-2.1	1.78			
N-stearoyl-sphinganine (d18:0/18:0)	Lipid	Sphingolipid Metabolism	0.0076	0.0473	-1.08	-3.43		5283573	
7-HOCA	Lipid	Sterol	0.0003	0.0046	-1.35	2.4	C17337	3081085	12458
ceramide (d16:1/24:1, d18:1/22:1)	Lipid	Ceramides	0.0003	0.0046	-1.93	2.38			
AMP	NT	Purine Metab, Adenine containing	0.0044	0.0319	1.19	-2.01	C00020	6083	00045
2'-O-methylcytidine	NT	Pyrimidine Metab, Cytidine containing	0.0032	0.0259	-2.27	1.22		150971	
alpha-CEHC sulfate	CF_Vit	Tocopherol Metabolism	0	0.0006	-2.81	1.26			
gamma-tocopherol/beta-tocopherol	CF_Vit	Tocopherol Metabolism	0.0002	0.0037	1.98	-2.13			
retinol (Vitamin A)	CF_Vit	Vitamin A Metabolism	0.0036	0.0284	-1.09	1.56	C00473	445354	00305
perfluorooctanesulfonic acid (PFOS)	Xeno	Chemical	0.0013	0.0133	-2.44	1.07	C18142	74483	59586
X– 11530		Unknown	0.0074	0.0471	-2.27	1.04			
X– 11538		Unknown	0.0014	0.0141	1.54	-1.67			
X– 11795		Unknown	0.0006	0.0077	4.09	2.14			
X– 12117		Unknown	0.0001	0.0033	1.11	2.6			
X– 13866		Unknown	0	0	-1.44	2.87			
X– 16580		Unknown	0.0003	0.0052	-1.15	-3.77			
X– 19141		Unknown	0.0002	0.0033	1.14	4.14			
X– 21785		Unknown	0.0011	0.0126	1.16	2.93			
X– 23369		Unknown	0.0043	0.0314	-1.59	1.48			
X– 23678		Unknown	0.0075	0.0471	1.17	-2			

SM, sphingomyelin; AA, amino acid; CHO, carbohydrate; NT, nucleotide; CF_Vit, cofactors and vitamins; Xeno, xenobiotic; FA, fatty acid; FDR, false discovery rate; FC, fold change; CON, control diet; CPB, cardiac protection blend. KEGG, Kyoto Encyclopedia of Genes and Genomes; PUBCHEM, open chemistry database at NIH; HMDB, Human Metabolome Database. Negative fold changes represent decreases in concentration from baseline to 6 months.

Ten metabolites with the highest VIP scores were identified using PLS-DA. Caprate, EPA, DHA and its phosphatidylcholine derivative DHA-PC, and X-13866, a metabolite with unknown identity were higher in CPB dogs than in CON dogs while two glycerophosphatidylcholines, one lysophospholipid, an unknown metabolite X-11795, and one sphingomyelin were lower in CPB dogs (Fig 1D). The values of R2 and Q2, estimates of model’s predictive accuracy, were reported in S3 File.

### Omega-6 to omega-3 FA ratio

**Fig 2:**
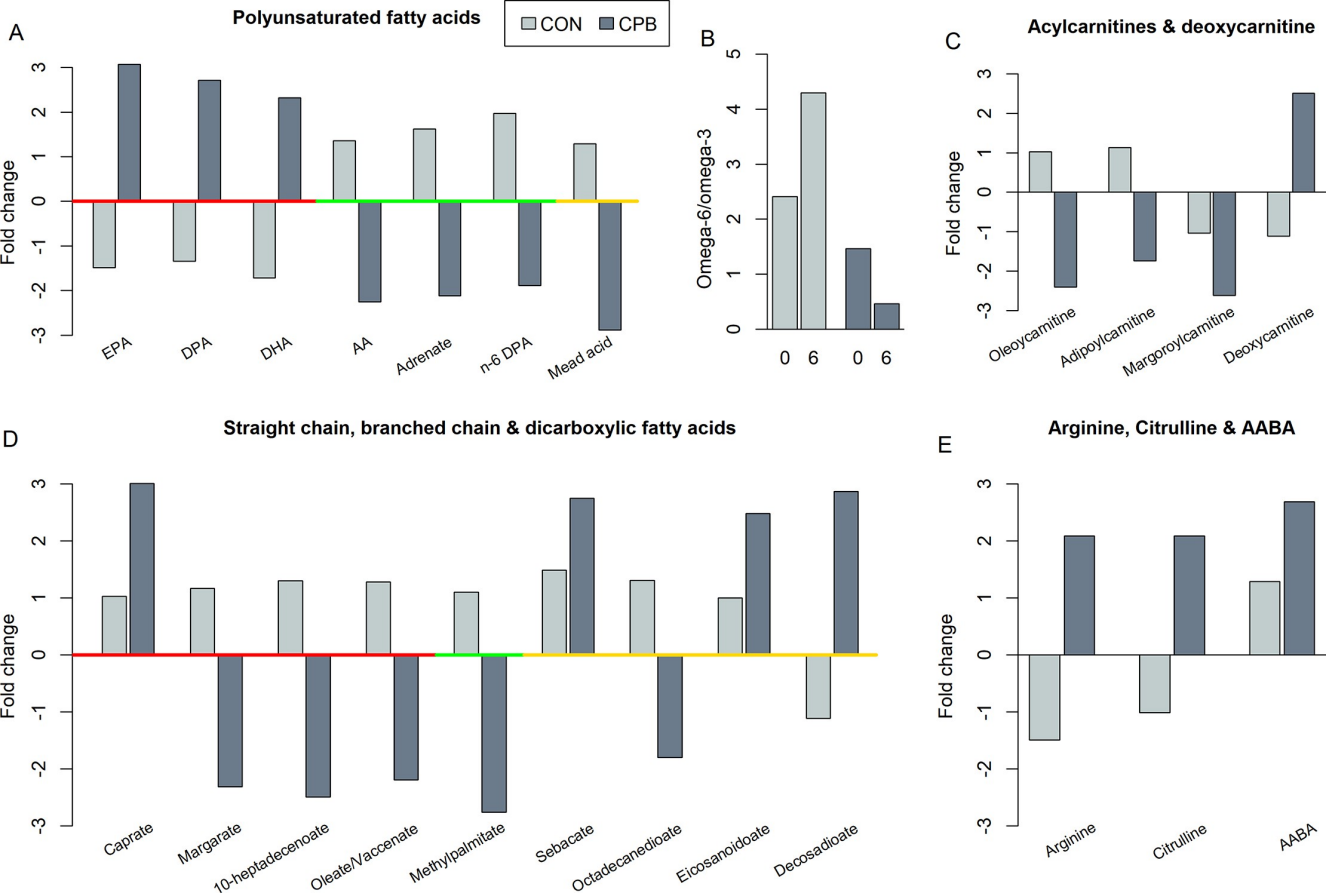
Fold changes between CPB vs. CON diets in (A) polyunsaturated FAs, (B) ratios of omega-6 to omega-3 FAs, (C) acylcarnitines and deoxycarnitine, (D) straight chain, branched chain, and dicarboxylic FAs, and (E) L-arginine, L-citrulline, and 2-aminobutyric acid (AABA) at baseline (0) and 6 months (6). Red, green, and gold bars along x-axis depict (A) omega-3, -6, and -9 FAs respectively and (D) straight, branched chain, and dicarboxylic FAs respectively. A negative fold change represents a decrease from baseline to 6 months.

Three omega-3 polyunsaturated fatty acids (PUFAs), eicosapentaenoate (EPA; 20:5n-3), docosapentaenoate (DPA; 22:5n-3), and docosahexaenoate (DHA; 22:6n-3) were increased in CPB dogs but decreased in CON dogs. In contrast, three omega-6 PUFAs, arachidonate (ARA, 20:4n-6), adrenate (22:4n-6), and docosapentaenoate (n6 DPA; 22:5n-6), and one omega-9 PUFA, mead acid (20:3n-9), were decreased over baseline in CPB dogs but increased in CON dogs (Fig 2A). At baseline, the ratio of serum omega-6 to omega-3 was 2.41 for CON dogs and 1.46 for CPB dogs. These ratios changed to 4.30 and 0.46 after 6 months respectively (Fig 2B).

### Acylcarnitines and deoxycarnitine

Two long chain acyl carnitines, oleoylcarnitine (C18) and margaroylcarnitine (C17), and a short chain dicarboxylic carnitine, adipoylcarnitine (C6-DC), showed large decreases from baseline to 6 months in CPB dogs (FCs = -2.41, -2.62 and -1.74 respectively, all FDR < 0.05. A negative FC indicates a decrease from baseline to 6 months) while little change was found in CON dogs (all FCs < 1.13) (Fig 2C). Two additional acylcarnitines, myristoleoylcarnitine (C14:1) and behenoylcarnitine (C22), were decreased in the CPB dogs although the changes did not reach statistical significance after p value adjustment (both P = 0.03, FDR = 0.12). The concentration of deoxycarnitine, the immediate precursor of carnitine biosynthesis, was significantly elevated in CPB dogs compared to CON dogs (FCs = 2.51 and -1.11 respectively, FDR = 0.004).

### MCFAs, LCFAs, BCFAs and DCFAs

Caprate, a 10-carbon MCFA, showed a significant increase from baseline in CPB dogs compared to little change in CON dogs (FDR < 0.001) (Fig 2D). Caprylate, an 8-carbon MCFA, showed a similar trend but the change did not reach statistical significance after p value adjustment (P = 0.025, FDR = 0.11, data not shown). Large decreases were observed in three LCFAs, margarate (17:0), 10-heptadecenoate (17:1n7), and oleate/vaccenate (18:1), and in a branched chain fatty acid (BCFA), the methyl ester of palmitic acid, in CPB dogs while little or no change was found in CON dogs (all FDR < 0.05, Fig 2D). Additionally, four dicarboxylic fatty acids (DCFAs) showed changes: sebacate, eicosanodioate, and decosadioate increased while octadecanedioate (C18) decreased in the CPB dogs (all FDR < 0.05, Fig 2D).

### Changes in amino acid profiles

On the pathway level, increases in lysine metabolism, phenylalanine and tyrosine metabolism, branch chain amino acid metabolism, and methionine, cysteine, and taurine metabolism were observed in CPB dogs vs. CON dogs (Table 1). Alpha-aminobutyric acid (AABA), a modulator of glutathione homeostasis in the myocardium [34], was increased by 2.69 fold in CPB dogs compared to a smaller 1.29-fold increase in CON dogs (Fig 2E, FDR = 0.026). Although arginine contents were similar between diets (CON: 1.53% vs. CPB: 1.50%, S1 Table), serum levels of arginine and citrulline were significantly elevated from baseline in CPB dogs (Fig 2E, both FC = 2.09, FDR = 0.031 and 0.024 respectively) but decreased in CON dogs.

### Phospholipids

**Fig 3:**
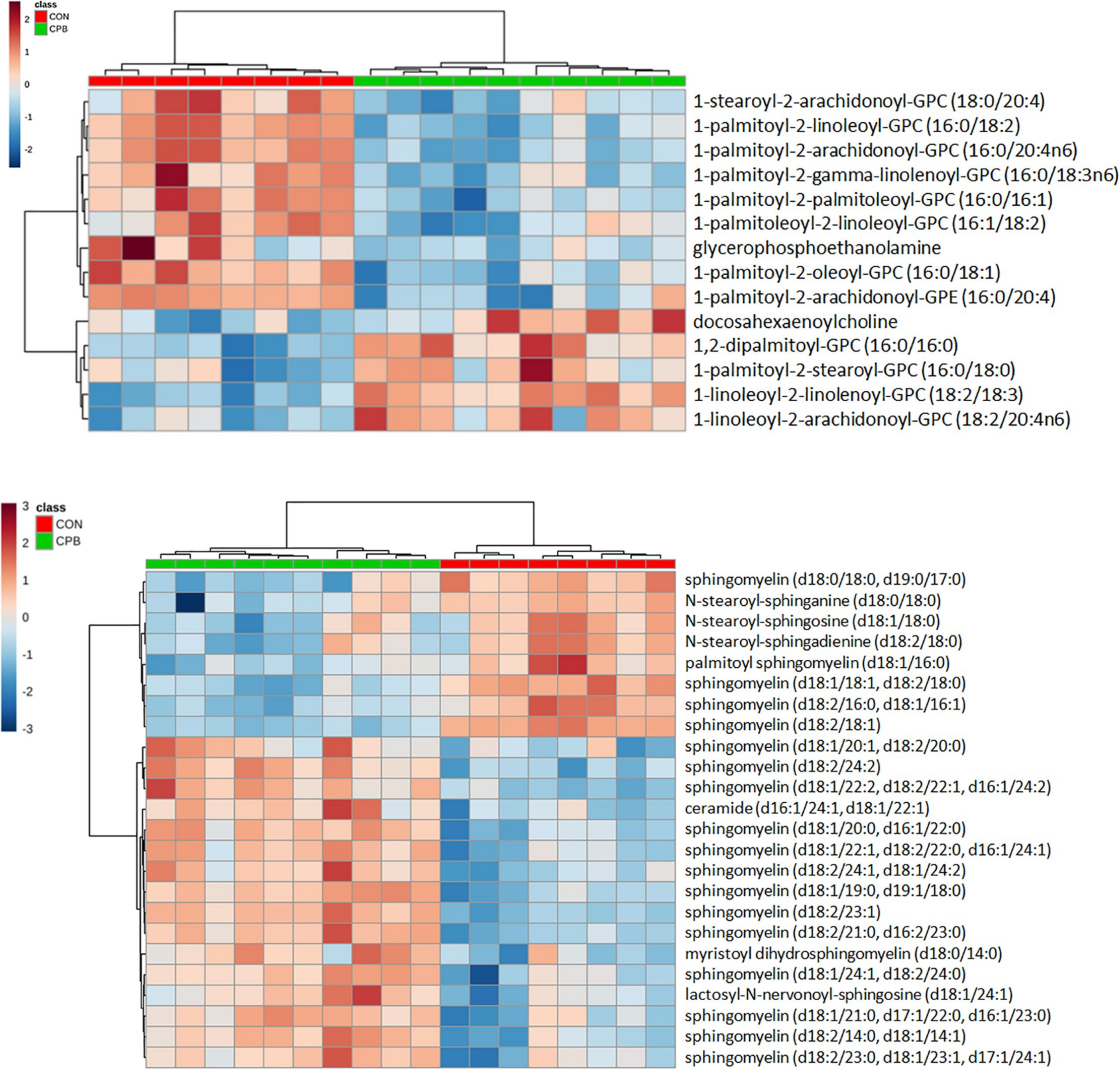
Heatmaps of changes of phospholipids (A) and sphingomyelins (B) from baseline to 6 months. Legends: diet groups are represented by red (CON) and green (CPB) boxes on the top of the heatmaps; Keys: orange indicates increases while blue indicates decreases from baseline.

Four glycerophosphatidyl cholines (GPCs) and DHA-choline were increased in CPB group vs. CON group while seven GPCs were decreased (Fig 3A). In addition, glycerophosphoethanolamine and 1-palmitoyl-2-arachidonoyl-glycerophosphatidyl ethanolamine (GPE) (16:0/20:4) were decreased (all FDR < 0.05).

Sphingomyelin (SM) is comprised of a phosphatidyl choline or phosphatidyl ethanolamine polar group linked to ceramide, which includes a sphingosine backbone and a FA. Overall, the majority (16/24) of differentially changed SMs were increased in CPB dogs when compared to CON dogs, resulting in an overall increase in SM in the serum (all FDR < 0.05, Fig 3B). SM was further examined based on the saturated LCFA constituents: palmitic acid (SM-16), arachidic acid (SM-20), behenic acid (SM-22), and lignoceric acid (SM-24). SM-16 was decreased by more than 3-fold in CPB dogs while SM-20, SM-22, and SM-24 were increased (Table 2). No significantly altered ceramide with saturated LCFA was found in this study.

**Table 2:** Sphingomyelin species with long chain saturated fatty acids.

Metabolite	Species	P-value	FDR	FC_CON	FC_CPB
palmitoyl SM (d18:1/16:0)	SM-16	0.001	0.011	-1.24	-3.14
SM (d18:2/16:0, d18:1/16:1)	SM-16	0.000	0.000	-1.09	-3.27
SM (d18:1/20:0, d16:1/22:0)	SM-20/22	0.000	0.001	-1.59	2.06
SM (d18:1/20:1, d18:2/20:0)	SM-20	0.008	0.047	-1.23	1.64
SM (d18:1/21:0, d17:1/22:0, d16:1/23:0)	SM-21/22/23	0.001	0.013	-1.57	1.97
SM (d18:1/22:1, d18:2/22:0, d16:1/24:1)	SM-22	0.000	0.003	-1.63	1.60
SM (d18:2/23:0, d18:1/23:1, d17:1/24:1)	SM-23	0.003	0.025	-1.54	1.79
SM (d18:1/24:1, d18:2/24:0)	SM-24	0.001	0.006	-1.54	1.93

SM, sphingomyelin; FDR, false discovery rate; FC, fold change; CON, control diet; CPB, cardiac protection blend.

Negative numbers (in green) denote decreases in concentration from baseline to 6 months while positive numbers

(in red) show increases

Plasmalogen is another unique group of phospholipids with a vinyl-ether bond in the 1-acyl position. Three of the identified plasmalogens were GPC while the other three belonged to GPE. The five plasmalogens with omega-6 PUFAs (arachidonoyl and linoleoyl) and omega-9 PUFA (oleoyl) in the 2-acyl position were decreased in CPB group whereas the one with a saturated palmitic acid (16:0) was increased (S2A–S2F Fig).

Lysolipids are derivatives of phospholipids in which 2-acyl groups are removed by hydrolysis. The concentrations of eight lysolipids, including four lysophosphatidyl cholines (LPC), three lysophosphatidyl ethanolamines, and one lysophosphatidyl inositol were altered: while 1-lignoceroyl-GPC (24:0) was increased (FDR = 0.004) in CPB dogs compared with CON dogs, seven others were decreased (all FDR < 0.05) (S2G–S2N Fig), resulting in a net decrease in circulating lysolipids.

### Correlations between LAD and metabolites

**Fig 4:**
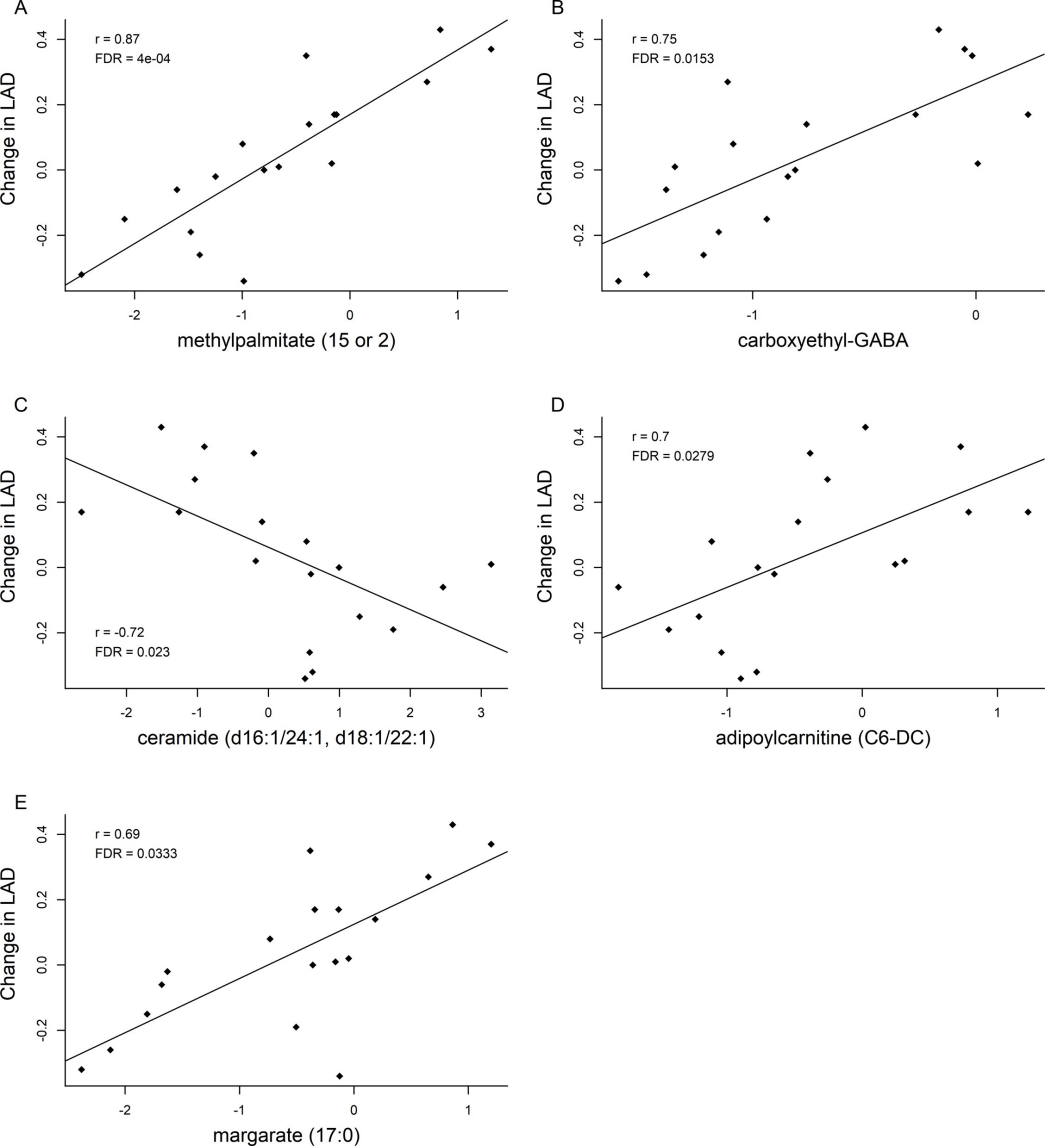
Scatterplots of changes between left atrial diameter (LAD) and (A) methylpalmitate, (B) carboxyethyl-GABA, (C) ceramide, (D) adipoylcarnitine, and (E) margarate. Correlation coefficients (r) and adjusted p-values (FDR) from Spearman’s correlation analysis are shown in the plots. A linear regression line is also drawn in each plot.

The 6-month changes of five metabolites were strongly correlated with changes in LAD (Fig 4, S2 Table). Methylpalmitate, carboxyethyl-GABA, adipoylcarnitine and margarate showed a positive correlation with LAD (FDR < 0.05, r > 0.68 in all cases), while ceramide had a negative correlation (FDR = 0.023, r = -0.72). Based on these results and previous finding that the levels of both methylpalmitate and margarate were decreased in MMVD dogs when compared to healthy dogs [9], the change in both of these 17-carbon FAs were evaluated relative to changes in other significant metabolites. Thirty-three metabolites were correlated with margarate and 57 were correlated with methylpalmitate (all r > 0.50, FDR < 0.05, S3 Table, S4 Table). Among them, 29 were in common (S5 Table), including positive correlations with 10-heptadecenoate (C17:1n7), mead acid, adrenate, and three acylcarnitines (adipoylcarnitine, margaroylcarnitine, and oleoylcarnitine) (S3 Fig, S4 Fig, all r > 0.59). Margarate and methylpalmitate had a nearly perfect correlation with each other (r = 0.91, FDR < 0.0001).

## Discussion

To our knowledge, this is the first study to investigate dietary effects on global metabolomic changes in dogs with a cardiac disease. Among the 102 known metabolites with significant diet by time interactions, approximately 72% were lipids and 16% were amino acids. Many of the observed changes were likely due to the differences between diets which differed considerably in their FA compositions. However, clinical benefits from the intervention study suggested a potential association between these diet-induced molecular and metabolic changes and clinical outcomes [17]. PCA analysis showed a clear clustering between diet groups after 6 months while no clustering was observed at baseline.

FAs are the main source of energy for the heart. Thus, lipid modification in the diet could have a primary effect on serum markers relating to cardiac energetics and offer potential benefits in dogs with MMVD by improving fat utilization in cardiac mitochondria. The two diets were similar in total fat content but differed in FA types. These differences were primarily the presence of both MCT and long chain omega-3 FAs in the CPB diet. Increases in serum capric acid (C10) (FDR < 0.001) and caprylic acid (C8) (FDR = 0.11) were seen in the CPB vs CON dogs reflecting this difference. The presence of these MCFAs can improve cardiac energetics and mitochondrial metabolism because MCFAs do not require transporters or carnitine-mediated transport pathway to reach mitochondria for oxidation [18, 19]. Studies have shown that MCTs produced more citric acid cycle intermediates and are more ketogenic than their long chain counterparts [19, 35, 36]. Importantly, MCTs reduced mitochondrial and cytoplasmic ROS in the rat liver [35] and in the heart of spontaneously hypertensive rats [20]. Three omega-3 PUFAs (EPA, DPA, and DHA) were increased in CPB dogs but decreased in CON dogs, as may be expected due to dietary differences. Conversely, three omega-6 PUFAs (arachidonate, adrenate, and n-6 DPA) were increased in CON dogs. Therefore, the ratios of omega-6 to omega-3 were 2.41 and 1.46 for CON and CPB groups at baseline but changed to 4.30 and 0.46 at 6 months respectively. Increases in the ratio of omega-6 to omega-3 are suggested to promote pathogenesis of many diseases including cardiovascular diseases, inflammation, and immune disorders while decreases in the ratio exert suppressive effects [37–39]. To the extent that increased serum MCFAs and omega-3 FAs are hallmarks of their cardiac bioavailability, improved myocardial energetics and protection against oxidative stress and inflammation appear to be facilitated by the CPB.

Acylcarnitines are intermediates of FA oxidation. Changes in circulating acylcarnitine concentrations have been used as diagnostic markers for disorders in peroxisomal or mitochondrial oxidation processes [40, 41]. Accumulation of acylcarnitine markers in the circulation likely signifies incomplete fat oxidation [41]. Both succinylcarnitine (C4-DC) and hexanoylcarnitine (C6:0) were increased in MMVD dogs when compared with healthy dogs [9]. In the present study, some carnitine metabolites, e.g., oleycarnitine (18:1), adipoylcarnitine (C6-DC), and margaroylcarnitine (17:0), were decreased in CPB dogs compared to CON dogs, suggesting an improvement in cardiac fat utilization. In addition, deoxycarnitine was increased in CPB dogs. In mammals, L-carnitine is synthesized in liver, brain, and (in humans) kidneys and is released into the circulatory system. The remaining organs are missing the hydroxylase which catalyzes the final conversion from deoxycarnitine to L-carnitine [42, 43]. Heart tissues do not synthesize L-carnitine but can generate deoxycarnitine, which can subsequently be exchanged for L-carnitine from the blood stream [42, 44]. Both in vivo and in vitro evidence demonstrated bidirectional exchanges between carnitine and deoxycarnitine across cardiac sarcolemma [42]. In dogs with MMVD, the myocardiocyte’s ability to synthesize deoxycarnitine may be compromised [9]. Hence, increases in serum deoxycarnitine associated with the CPB diet likely indicate a pathway for the heart to refresh its L-carnitine supply and promote further mitochondrial fat oxidation.

The serum levels of four dicarboxylic fatty acids (DCFAs) differed between diet groups. Sebacate (C10), eicosanodioate (C20:5n-3), and docosadioate (C22:6n-3) were increased in the CPB dogs vs CON dogs while octadecanedioate (C18) showed the opposite. The change in octadecanedioate may be explained by the fact that there was three times more stearic acid (C18:0) in CON diet versus CPB diet (CON: 1.85% vs. CPB: 0.58%, S1 Table). In early studies, DCFAs were found in urine from healthy individuals after administration of MCTs [45]. DCFAs are the products of monocarboxylic ω-oxidation in peroxisomes, which under normal conditions does not appear to be a major pathway. However, this pathway has been proposed as a rescue pathway where mitochondrial dysfunction exists [46]. The results from the present study suggest a potential involvement of MCT-DCFA-ω-oxidation rescue pathway in improving energy metabolism in MMVD dogs with compromised mitochondrial function.

L-arginine concentrations were similar between diets. However, a two-fold increase in serum arginine and citrulline were found in CPB dogs. Nitric oxide (NO) exerts many beneficial roles in the cardiovascular system and protects against hypertension, oxidative stress and cell death [47–50]. Nitric oxide is synthesized by nitric oxide synthase which converts L-arginine to NO and L-citrulline. Supplementation of L-arginine plus L-citrulline have been shown to cause a more rapid increase in NO bioavailability and NO-dependent effects than with each amino acid alone [51]. It was previously noted that, there was an increase of oxidized glutathione but decrease of reduced glutathione in MMVD dogs when compared to healthy dogs [9, 52]. AABA, a modulator for myocardial glutathione homeostasis, was increased 2.7 fold in CPB dogs. AABA activates the intracellular glutathione biosynthesis pathway, rendering protective effects against ROS in murine cardiomyopathy model [34]. Data from the current study suggests that the CPB may also confer NO-dependent protection on the cardiovascular systems and ameliorate oxidative stress via glutathione biosynthesis.

Many of the changes in phospholipids were likely due to the difference in diets relating to FAs. One interesting finding, however, was the decrease in 1-palmitoyl-2-arachidonoyl-GPE (16:0/20:4), in CPB dogs. Elevated phosphatidyl ethanolamine has been previously associated with occlusive arterial disease in human [53]. While not conclusive, these data support the possibility that the decrease in phosphatidyl ethanolamine is associated with improved cardiac function in MMVD-CPB dogs. Lysolipids are the hydrolytic product of phospholipase A_1_, resulting in respective 1-acyl phospholipids. Plasmalogens belong to a unique group of phospholipids with a vinyl ether bond in the 1-acyl position and commonly a polyunsaturated FA in the 2-acyl position. Plasmalogens constitute 10% of the total phospholipid molecular mass and more than 30% of phospholipids in the adult human heart [54]. In dogs, over 50% of phospholipids in the myocardial sarcolemma are plasmalogens [55]. Both lysolipids and plasmalogens are involved in cellular signaling pathways. In the present study, seven out of eight lysolipids and five out of six plasmalogens were decreased in CPB-fed dogs vs. CON-fed dogs. The biological significance of this, if any, remains to be determined.

Ceramides (Cer) and sphingomyelin (SM) exhibit many biological activities that may influence the pathophysiology of heart failure [56–58]. These properties differ, depending on the LCFA attached. Recent studies suggested causal associations between levels of Cer and SM species with long chain saturated FAs and risk of heart failure. In a meta-analysis with nearly 6000 human participants, the ratio of C24:0 containing ceramide to C16:0 ceramide were inversely associated with coronary heart disease and incident heart failure [56]. Plasma phospholipid with very long chain saturated FAs were associated with lower risk of incident heart failure [57]. In further support of this association, a more recent study with over 4000 participants and a median follow-up of 9.4 years documented that higher levels of Cer-16 and SM-16 with palmitic acid were associated with increased risk of heart failure while higher levels of SM-20, Cer-22, SM-22, and SM-24 were associated with decreased risk of heart failure [58]. Unlike these human studies, Cer with saturated LCFA did not vary within this present study in dogs. However, the levels of SM-16 were lower while levels of SM-20, SM-22, and SM-24 were higher in CPB dogs compared to CON dogs. Hence, while the cardiac diseases in the human studies and this canine study differ, the observed changes in the levels and FA constituents of circulating SMs may suggest markers of cardiac health in dogs and the CPB diet may have thus contributed to a degree of cardiac protection in canine MMVD through these metabolites.

Although even chain FAs represent >99% of the total circulating FAs, there are also detectable amounts of odd-chain FAs in human tissues [59, 60]. Several recent studies have associated plasma odd chain saturated FAs, mainly C15:0 and C17:0, with reduced risk of cardiometabolic diseases [60–63]. Methylpalmitate, a palmitic acid (C16:0) methyl ester, has been associated with anti-inflammatory and anti-oxidative activities, and may possess cardioprotective and antifibrotic effects [64–67]. However, a previous study reported that serum concentrations of margarate and methylpalmitate were increased in dogs with early stage MMVD when compared to healthy dogs [9]. This may suggest a species difference in dogs versus humans. Importantly, in the present study, both margarate and methylpalmitate were decreased in response to the diet intervention yet these decreases were positively associated with reductions in LAD in preclinical MMVD dogs. Because little is known about margarate or methylpalmitate in cardiac health in dogs, more studies from larger cohorts of dogs are warranted.

In summary, untargeted serum metabolomic analysis has identified numerous metabolites that may reflect improved cardiac bioenergetics and fatty acid utilization by cardiac mitochondria in dogs fed the CPB diet. Metabolomic markers also suggest improved cellular redox state and reduced inflammation in MMVD dogs fed the CPB diet. Due to the many similarities between human and canine MMVD, our study may also shed light on metabolic perturbations in human MMVD.

## Supporting information

S1 Table Amino acid and fatty acid analysis of CON and CPB diets. (DOCX)

S2 Table Spearman’s correlation analysis on changes between LAD and significant metabolites. (DOCX)

S3 Table Spearman’s correlation analysis on changes between margarate and other significant metabolites. (DOCX)

S4 Table Spearman’s correlation analysis on changes between methylpalmitate and other significant metabolites. (DOCX)

S5 Table Metabolites that are significantly correlated with both margarate and methylpalmitate. (DOCX)

S1 Fig PCA plots on time effects in CON and CPB diets. (TIF)

S2 Fig Diet by time interaction plots for plasmalogens and lysophospholipids. (TIF)

S3 Fig Scatterplots of changes between margarate and seven other metabolites. (TIFF)

S4 Fig Scatterplots of changes between methylpalmitate and seven other metabolites. (TIFF)

S1 File Supplemental methods. (DOCX)

S2 File Raw data. (XLSX)

S3 File Multiple linear regression and PLS-DA analysis. (DOCX)

## Abbreviations

CPB: cardiac protection blend
CON: control diet
MMVD: myxomatous mitral valve disease
LAD: left atrial diameter
FDR: false discovery rate
PCA: principal component analysis
MCT: medium chain triglycerides
FA: fatty acid
EPA: eicosapentaenoate (20:5n-3)
DHA: docosahexaenoate (22:6n-3)
DPA: docosapentaenoate (22:5n-3)
MCFA: medium chain fatty acid
LCFA: long chain fatty acid
BCFA: branched chain fatty acid
PUFA: polyunsaturated fatty acid
DCFA: dicarboxylic fatty acid
SM: sphingomyelin
Cer: ceramide
GPC: glycerophosphatidyl choline
GPE: glycerophosphatidyl ethanolamine
LPC: lysophosphatidyl choline
AABA: α-aminobutyric acid or 2-aminobutyric acid
ROS: reactive oxygen species
FC: fold change
