# Supplementary material for: Serum untargeted metabolomic changes in response to diet intervention in dogs with preclinical myxomatous mitral valve disease
Source: PLoS One. 2020 Jun 18;15(6):e0234404. doi: 10.1371/journal.pone.0234404 (PMC7302913; doi:10.1371/journal.pone.0234404)
Supplement: S1 Table — (DOCX) [file pone.0234404.s001.docx]

**S1 Table.** Amino acid and fatty acid analysis of diets.

|  | **CON** | | **CPB** | |
| --- | --- | --- | --- | --- |
| Nutrient | **DM basis** | **Calorie basis** | **DM basis** | **Calorie basis** |
|  | % | g/100Kcal ME | % | g/100Kcal ME |
| Essential Amino Acids |  |  |  |  |
| Arginine | 1.53 | 0.38 | 1.50 | 0.38 |
| Histidine | 0.62 | 0.16 | 0.60 | 0.15 |
| Isoleucine | 1.09 | 0.27 | 1.01 | 0.26 |
| Leucine | 2.78 | 0.69 | 2.56 | 0.66 |
| Lysine | 1.16 | 0.29 | 2.12 | 0.54 |
| Methionine | 0.62 | 0.16 | 1.45 | 0.37 |
| Phenylalanine | 1.40 | 0.35 | 1.29 | 0.33 |
| Threonine | 1.03 | 0.26 | 0.99 | 0.25 |
| Tryptophan | 0.22 | 0.06 | 0.22 | 0.06 |
| Valine | 1.41 | 0.35 | 1.33 | 0.34 |
|  |  |  |  |  |
| Other Amino Acids |  |  |  |  |
| Alanine | 1.98 | 0.49 | 1.88 | 0.48 |
| Aspartic Acid | 2.09 | 0.52 | 2.00 | 0.51 |
| Cysteine | 0.46 | 0.11 | 0.42 | 0.11 |
| Glutamic Acid | 5.12 | 1.28 | 4.83 | 1.24 |
| Glycine | 1.73 | 0.43 | 1.75 | 0.45 |
| Proline | 2.06 | 0.51 | 1.92 | 0.49 |
| Serine | 1.32 | 0.33 | 1.23 | 0.32 |
| Taurine | 693.08 | 17.30 | 2035.93 | 52.21 |
| Tyrosine | 0.99 | 0.25 | 0.93 | 0.24 |
|  |  |  |  |  |
| Fatty Acids |  |  |  |  |
|  | % | mg/100kcal | % | mg/100kcal |
| C8:0 Caprylic | BDL* | BDL | 1.27 | 325.85 |
| C10:0 Capric | 0.02 | 4.76 | 1.17 | 298.80 |
| C12:0 Lauric | BDL | BDL | 0.02 | 4.13 |
| C14:0 Myristic | 0.27 | 66.15 | 0.31 | 78.46 |
| C14:1n5 Myristoleic | 0.05 | 13.35 | 0.03 | 7.73 |
| C15:0 Pentadecanoic | 0.04 | 10.24 | 0.03 | 7.14 |
| C16:0 Palmitic | 3.65 | 911.91 | 2.34 | 601.35 |
| C16:1n7 Palmitoleic | 0.54 | 135.07 | 0.66 | 169.22 |
| C16:2 Hexadecadienoic | BDL | BDL | 0.04 | 10.48 |
| C16:3n4 Hexadecatrienoic | BDL | BDL | 0.06 | 14.76 |
| C17:0 Margaric | 0.11 | 27.16 | 0.03 | 7.85 |
| C18:0 Stearic | 1.85 | 461.24 | 0.58 | 148.73 |
| C18:1n7C Vaccenic | 0.25 | 62.97 | 0.23 | 58.85 |
| C18:1n9C Oleic | 5.33 | 1329.54 | 2.84 | 727.24 |
| C18:1n9T Elaidic | 0.40 | 100.38 | 0.06 | 14.46 |
| C18:2 Other trans Isomers | 0.04 | 10.77 | 0.04 | 9.35 |
| C18:2n6 Linoleic | 2.63 | 655.52 | 2.45 | 628.87 |
| C18:3n3 Linolenic | 0.12 | 30.79 | 0.16 | 40.87 |
| C18:3n6 Gamma Linolenic | BDL | BDL | 0.02 | 5.33 |
| C18:4n3 Octadecatetraenoic | BDL | BDL | 0.07 | 18.62 |
| C20:0 Arachidic | 0.02 | 5.62 | 0.02 | 5.56 |
| C20:1n9 cis Eicosenoic | 0.04 | 9.58 | 0.06 | 15.17 |
| C20:2n6 Eicosadienoic | BDL | BDL | 0.02 | 6.27 |
| C20:3n6 Homo-Gamma-Linolenic | 0.02 | 4.26 | 0.02 | 5.39 |
| C20:4n3 Eicosatetraenoic | BDL | BDL | 0.04 | 9.60 |
| C20:4n6 Arachidonic | 0.06 | 16.06 | 0.09 | 23.19 |
| C20:5n3 Eicosapentaenoic | 0.03 | 7.53 | 0.42 | 106.98 |
| C21:0 Heneicosanoic | 0.03 | 8.33 | BDL | BDL |
| C21:5 Heneicosapentaenoic | BDL | BDL | 0.02 | 4.39 |
| C22:0 Behenic | BDL | BDL | 0.01 | 3.51 |
| C22:5n3 Docosapentaenoic | BDL | BDL | 0.08 | 20.38 |
| C22:5n6 Docosapentaenoic | BDL | BDL | 0.02 | 4.98 |
| C22:6n3 Docosahexaenoic | 0.03 | 6.34 | 0.30 | 76.59 |
| C24:1n9 Nervonic | BDL | BDL | 0.02 | 4.57 |

BDL* = Below detection limits
