# Supplementary material for: Serum untargeted metabolomic changes in response to diet intervention in dogs with preclinical myxomatous mitral valve disease
Source: PLoS One. 2020 Jun 18;15(6):e0234404. doi: 10.1371/journal.pone.0234404 (PMC7302913; doi:10.1371/journal.pone.0234404)
Supplement: S2 Table — (DOCX) [file pone.0234404.s002.docx]

**S2 Table**. Spearman’s correlation analysis on changes between LAD and significant metabolites.

| Metabolites | p-val | fdr | r |
| --- | --- | --- | --- |
| methylpalmitate (15 or 2) | 3.55E-06 | 0.000358 | 0.865256 |
| carboxyethyl-GABA | 0.000303 | 0.015316 | 0.753743 |
| ceramide (d16:1/24:1, d18:1/22:1) | 0.000684 | 0.02304 | -0.7238 |
| adipoylcarnitine (C6-DC) | 0.001106 | 0.027925 | 0.704182 |
| margarate (17:0) | 0.001648 | 0.03329 | 0.686629 |
