# Supplementary material for: Serum untargeted metabolomic changes in response to diet intervention in dogs with preclinical myxomatous mitral valve disease
Source: PLoS One. 2020 Jun 18;15(6):e0234404. doi: 10.1371/journal.pone.0234404 (PMC7302913; doi:10.1371/journal.pone.0234404)
Supplement: S3 Table — (DOCX) [file pone.0234404.s003.docx]

**S3 Table**. Spearman’s correlation analysis on changes between margarate and other significant

metabolites.

| Metabolites | r | p-val | fdr |
| --- | --- | --- | --- |
| sphingomyelin (d18:1/22:2, d18:2/22:1, d16:1/24:2) | -0.68 | 0.0021 | 0.0329 |
| docosahexaenoylcholine | -0.66 | 0.003 | 0.0353 |
| cystathionine | -0.64 | 0.0043 | 0.0353 |
| S-methylmethionine | -0.62 | 0.006 | 0.0375 |
| 1,2-dipalmitoyl-GPC (16:0/16:0) | -0.61 | 0.0071 | 0.0411 |
| 2'-O-methylcytidine | -0.58 | 0.0117 | 0.0472 |
| 1-linoleoyl-2-linolenoyl-GPC (18:2/18:3) | -0.57 | 0.0133 | 0.0472 |
| 1-palmitoyl-2-stearoyl-GPC (16:0/18:0) | -0.57 | 0.0127 | 0.0472 |
| myristoyl dihydrosphingomyelin (d18:0/14:0) | -0.57 | 0.0144 | 0.0472 |
| arginine | -0.56 | 0.0148 | 0.0472 |
| 1-(1-enyl-palmitoyl)-2-palmitoyl-GPC (P-16:0/16:0) | -0.56 | 0.0151 | 0.0472 |
| sphingomyelin (d18:2/23:1) | -0.56 | 0.015 | 0.0472 |
| 1-palmitoyl-2-arachidonoyl-GPC (16:0/20:4n6) | 0.56 | 0.0148 | 0.0472 |
| N-stearoyl-sphingadienine (d18:2/18:0) | 0.56 | 0.0163 | 0.0494 |
| 1-(1-enyl-stearoyl)-2-arachidonoyl-GPE (P-18:0/20:4) | 0.57 | 0.014 | 0.0472 |
| 1-stearoyl-2-arachidonoyl-GPC (18:0/20:4) | 0.58 | 0.0123 | 0.0472 |
| adipoylcarnitine (C6-DC) | 0.59 | 0.0097 | 0.0459 |
| 1-linoleoyl-GPE (18:2) | 0.59 | 0.0101 | 0.0459 |
| 1-palmitoyl-2-palmitoleoyl-GPC (16:0/16:1) | 0.6 | 0.0085 | 0.044 |
| sphingomyelin (d18:2/16:0, d18:1/16:1) | 0.6 | 0.0088 | 0.044 |
| 1-(1-enyl-palmitoyl)-2-arachidonoyl-GPC (P-16:0/20:4) | 0.61 | 0.0074 | 0.0411 |
| methionine sulfone | 0.63 | 0.0052 | 0.0353 |
| 1-palmitoleoyl-2-linoleoyl-GPC (16:1/18:2) | 0.63 | 0.005 | 0.0353 |
| sphingomyelin (d18:1/18:1, d18:2/18:0) | 0.63 | 0.0048 | 0.0353 |
| sphingomyelin (d18:2/18:1) | 0.63 | 0.0053 | 0.0353 |
| margaroylcarnitine | 0.65 | 0.0037 | 0.0353 |
| 1-(1-enyl-palmitoyl)-2-arachidonoyl-GPE (P-16:0/20:4) | 0.65 | 0.0033 | 0.0353 |
| oleoylcarnitine (C18) | 0.67 | 0.0022 | 0.0329 |
| 1-arachidonoyl-GPE (20:4) | 0.67 | 0.0023 | 0.0329 |
| adrenate (22:4n6) | 0.7 | 0.0011 | 0.0329 |
| mead acid (20:3n9) | 0.7 | 0.0014 | 0.0329 |
| 10-heptadecenoate (17:1n7) | 0.78 | 0.0002 | 0.01 |
| methylpalmitate (15 or 2) | 0.91 | 0 | 0 |
