# Supplementary material for: Serum untargeted metabolomic changes in response to diet intervention in dogs with preclinical myxomatous mitral valve disease
Source: PLoS One. 2020 Jun 18;15(6):e0234404. doi: 10.1371/journal.pone.0234404 (PMC7302913; doi:10.1371/journal.pone.0234404)
Supplement: S4 Table — (DOCX) [file pone.0234404.s004.docx]

**S4 Table.** Spearman’s correlation analysis on changes between methylpalmitate and other significant metabolites.

| Metabolites | r | p-val | fdr |
| --- | --- | --- | --- |
| 1,2-dipalmitoyl-GPC (16:0/16:0) | -0.71 | 0.0009 | 0.015 |
| sphingomyelin (d18:1/22:2, d18:2/22:1, d16:1/24:2) | -0.71 | 0.001 | 0.015 |
| 1-linoleoyl-2-linolenoyl-GPC (18:2/18:3) | -0.7 | 0.0014 | 0.015 |
| sphingomyelin (d18:1/20:0, d16:1/22:0) | -0.69 | 0.0017 | 0.015 |
| sphingomyelin (d18:2/23:1) | -0.69 | 0.0017 | 0.015 |
| 1-palmitoyl-2-stearoyl-GPC (16:0/18:0) | -0.67 | 0.0024 | 0.0152 |
| sphingomyelin (d18:1/22:1, d18:2/22:0, d16:1/24:1) | -0.67 | 0.0021 | 0.0152 |
| cystathionine | -0.66 | 0.0026 | 0.0152 |
| myristoyl dihydrosphingomyelin (d18:0/14:0) | -0.66 | 0.003 | 0.0152 |
| sphingomyelin (d18:2/14:0, d18:1/14:1) | -0.66 | 0.003 | 0.0152 |
| sphingomyelin (d18:1/19:0, d19:1/18:0) | -0.65 | 0.0034 | 0.0152 |
| 1-lignoceroyl-GPC (24:0) | -0.64 | 0.0045 | 0.0161 |
| sphingomyelin (d18:1/21:0, d17:1/22:0, d16:1/23:0) | -0.64 | 0.0041 | 0.0152 |
| ceramide (d16:1/24:1, d18:1/22:1) | -0.64 | 0.0041 | 0.0152 |
| sphingomyelin (d18:2/21:0, d16:2/23:0) | -0.63 | 0.0055 | 0.019 |
| eicosapentaenoate (EPA; 20:5n3) | -0.59 | 0.0095 | 0.0288 |
| hypotaurine | -0.58 | 0.0113 | 0.0323 |
| N-acetylphenylalanine | -0.57 | 0.0128 | 0.0335 |
| S-methylmethionine | -0.57 | 0.0134 | 0.0335 |
| docosahexaenoylcholine | -0.57 | 0.014 | 0.0341 |
| 2'-O-methylcytidine | -0.57 | 0.0133 | 0.0335 |
| sphingomyelin (d18:1/24:1, d18:2/24:0) | -0.56 | 0.0164 | 0.0373 |
| sphingomyelin (d18:2/24:1, d18:1/24:2) | -0.55 | 0.0179 | 0.0389 |
| 1-linoleoyl-2-arachidonoyl-GPC (18:2/20:4n6) | -0.54 | 0.0213 | 0.0422 |
| sphingomyelin (d18:2/24:2) | -0.54 | 0.0195 | 0.0406 |
| caprate (10:0) | -0.53 | 0.0226 | 0.0422 |
| lactosyl-N-nervonoyl-sphingosine (d18:1/24:1) | -0.53 | 0.0241 | 0.0438 |
| retinol (Vitamin A) | -0.53 | 0.0228 | 0.0422 |
| 1-palmitoyl-2-linoleoyl-GPC (16:0/18:2) | 0.52 | 0.0273 | 0.0479 |
| 1-(1-enyl-stearoyl)-2-oleoyl-GPE (P-18:0/18:1) | 0.52 | 0.0264 | 0.0471 |
| 1-stearoyl-2-arachidonoyl-GPC (18:0/20:4) | 0.53 | 0.0222 | 0.0422 |
| 1-palmitoleoyl-2-linoleoyl-GPC (16:1/18:2) | 0.54 | 0.0216 | 0.0422 |
| sphingomyelin (d18:0/18:0, d19:0/17:0) | 0.54 | 0.0205 | 0.0418 |
| docosapentaenoate (n6 DPA; 22:5n6) | 0.55 | 0.0177 | 0.0389 |
| 1-linoleoyl-GPE (18:2) | 0.55 | 0.0192 | 0.0406 |
| 1-palmitoyl-2-arachidonoyl-GPC (16:0/20:4n6) | 0.56 | 0.0157 | 0.0373 |
| 1-(1-enyl-palmitoyl)-2-linoleoyl-GPC (P-16:0/18:2) | 0.56 | 0.0163 | 0.0373 |
| sphingomyelin (d18:2/16:0, d18:1/16:1) | 0.57 | 0.0131 | 0.0335 |
| 1-palmitoyl-2-palmitoleoyl-GPC (16:0/16:1) | 0.58 | 0.0109 | 0.0321 |
| gamma-tocopherol/beta-tocopherol | 0.58 | 0.0125 | 0.0335 |
| 1-(1-enyl-stearoyl)-2-arachidonoyl-GPE (P-18:0/20:4) | 0.59 | 0.0093 | 0.0288 |
| 1-palmitoyl-2-gamma-linolenoyl-GPC (16:0/18:3n6) | 0.61 | 0.0074 | 0.0239 |
| 1-arachidonoyl-GPI (20:4) | 0.62 | 0.0062 | 0.0207 |
| carboxyethyl-GABA | 0.64 | 0.004 | 0.0152 |
| oleoylcarnitine (C18) | 0.65 | 0.0038 | 0.0152 |
| margaroylcarnitine | 0.65 | 0.0036 | 0.0152 |
| 1-(1-enyl-palmitoyl)-2-arachidonoyl-GPE (P-16:0/20:4) | 0.65 | 0.0035 | 0.0152 |
| sphingomyelin (d18:2/18:1) | 0.65 | 0.0032 | 0.0152 |
| 1-(1-enyl-palmitoyl)-2-arachidonoyl-GPC (P-16:0/20:4) | 0.66 | 0.0029 | 0.0152 |
| methionine sulfone | 0.67 | 0.0022 | 0.0152 |
| adrenate (22:4n6) | 0.68 | 0.0017 | 0.015 |
| mead acid (20:3n9) | 0.68 | 0.0018 | 0.015 |
| 1-arachidonoyl-GPE (20:4) | 0.69 | 0.0015 | 0.015 |
| sphingomyelin (d18:1/18:1, d18:2/18:0) | 0.69 | 0.0016 | 0.015 |
| adipoylcarnitine (C6-DC) | 0.71 | 0.001 | 0.015 |
| 10-heptadecenoate (17:1n7) | 0.78 | 0.0001 | 0.005 |
| margarate (17:0) | 0.91 | 0 | 0 |
