# Supplementary figures and images for: Serum untargeted metabolomic changes in response to diet intervention in dogs with preclinical myxomatous mitral valve disease
Source: PLoS One. 2020 Jun 18;15(6):e0234404. doi: 10.1371/journal.pone.0234404 (PMC7302913; doi:10.1371/journal.pone.0234404)

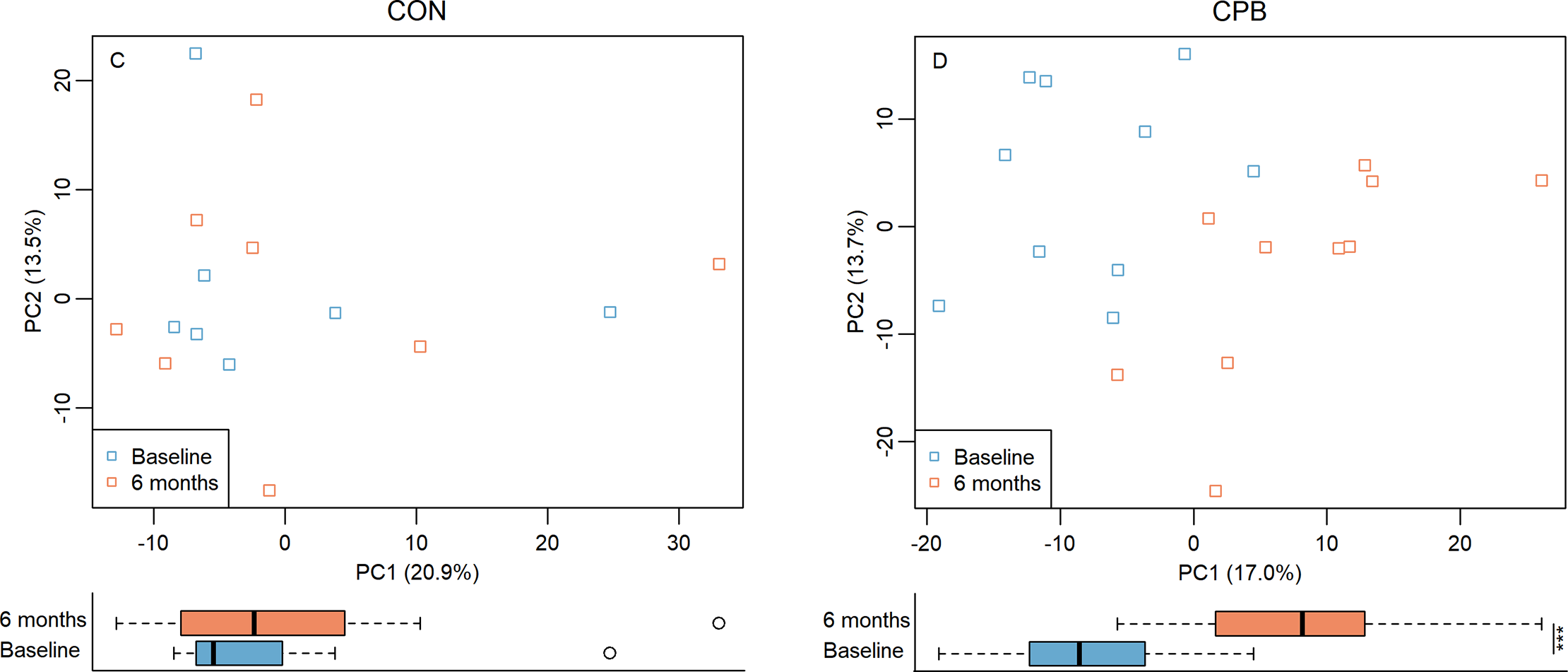

Supplement: S1 Fig — (TIF) [file pone.0234404.s006.tif]

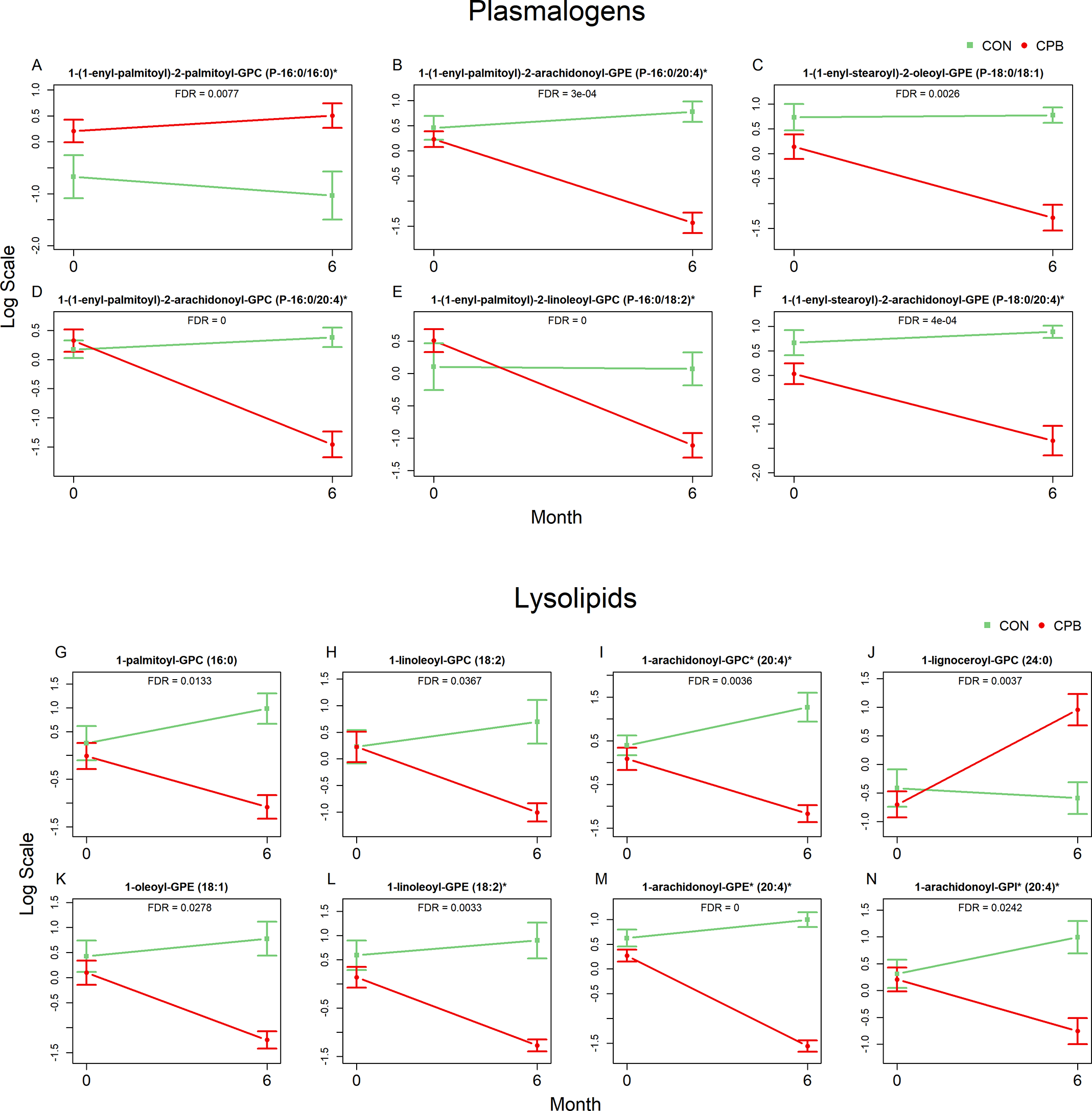

Supplement: S2 Fig — (TIF) [file pone.0234404.s007.tif]
