# Supplementary material for: Serum untargeted metabolomic changes in response to diet intervention in dogs with preclinical myxomatous mitral valve disease
Source: PLoS One. 2020 Jun 18;15(6):e0234404. doi: 10.1371/journal.pone.0234404 (PMC7302913; doi:10.1371/journal.pone.0234404)
Supplement: S3 File — (DOCX) [file pone.0234404.s012.docx]

**A. Multiple linear regressions of the 1^st^ principal components (PC1) on diet, adjusted for age, breed, sex and BCS.**

Baseline data

For PC1, no variable had a significant confounding effect (P > 0.05).

Multiple R-squared = 0.43

Adjusted R-squared = 0.19

Model p-value = 0.19

6 months data

For PC1, BCS was the only significant variable (P = 0.041). Neither age, sex nor breed had a significant confounding effect (P > 0.05).

Multiple R-squared = 0.85

Adjusted R-squared = 0.78

Model p-value = 1.54e-04

**B. R2 and Q2 values from the PLS-DA analysis at 6 months.**

The optimal number of 3 components achieved the highest predictive ability of the model.

**
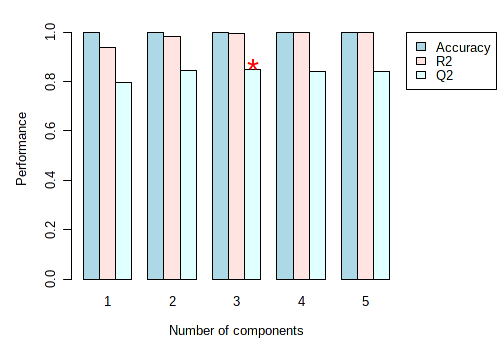
**
